# Novel Phenotypic Insights into the *IDS* c.817C>T Variant in Mucopolysaccharidosis Type II from Newborn Screening Cohorts

**DOI:** 10.3390/ijns11030068

**Published:** 2025-08-26

**Authors:** Éliane Beauregard-Lacroix, Caitlin Menello, Madeline Steffensen, Hsiang-Yu Lin, Chih-Kuang Chuang, Shuan-Pei Lin, Can Ficicioglu

**Affiliations:** 1 Section of Biochemical Genetics, Division of Human Genetics, Children’s Hospital of Philadelphia, Philadelphia, PA 19104, USA; eliane.beauregard-lacroix@umontreal.ca (É.B.-L.); menelloc@chop.edu (C.M.); steffensem@chop.edu (M.S.); 2 Department of Pediatrics, Medical Research and International Rare Disease Center, MacKay Memorial Hospital, Taipei 10449, Taiwan; lxc46199@ms37.hinet.net (H.-Y.L.); mmhcck@gmail.com (C.-K.C.); 4535lin@gmail.com (S.-P.L.); 3 Department of Medicine, MacKay Medical University, New Taipei City 25245, Taiwan; 4 Department of Pediatrics, Perelman School of Medicine, University of Pennsylvania, Philadelphia, PA 19104, USA

**Keywords:** mucopolysaccharidosis type II, Hunter syndrome, lysosomal storage disease, newborn screening

## Abstract

Mucopolysaccharidosis (MPS) type II, or Hunter syndrome, is an X-linked lysosomal storage disorder caused by a deficiency of iduronate-2-sulfatase. Glycosaminoglycan (GAG) accumulation leads to progressive multisystemic involvement, with coarse facial features, hepatosplenomegaly, short stature, recurrent upper respiratory infections, hearing loss, hernias, dysostosis multiplex, joint contractures, and cardiac valve disease. Individuals with the neuronopathic form of the disease also have central nervous system (CNS) involvement with developmental delay and progressive cognitive decline. Enzyme replacement therapy (ERT), idursulfase, is the only FDA-approved treatment for MPS II. MPS II was added to the Recommended Uniform Screening Panel (RUSP) in the United States in 2022, and screening is ongoing in several other countries, including Taiwan. Here, we report seven individuals from four families identified through newborn screening sharing the same *IDS* variant: c.817C>T, p.Arg273Trp. Confirmatory testing demonstrated low iduronate-2-sulfatase activity level and elevated GAGs in every individual, but they had no signs or symptoms of MPS II. They were aged 8 months to 60 years old according to the most recent assessment and all remained asymptomatic. ERT was not initiated for any of them. Our findings suggest that the *IDS* c.817C>T variant is associated with abnormal biochemical findings but no clinical phenotype of MPS II. Newborn screening will likely identify additional cases and provide a better understanding of the clinical significance of this variant.

## 1. Introduction

Mucopolysaccharidosis (MPS) type II, or Hunter syndrome, is an X-linked lysosomal storage disorder (LSD) caused by a deficiency of iduronate-2-sulfatase. This enzyme is encoded by the *IDS* gene and is involved in the degradation of glycosaminoglycans (GAGs). Its deficiency leads to the accumulation of heparan sulfate and dermatan sulfate within lysosomes. MPS II is characterized by progressive multisystemic involvement, with coarse facial features, hepatosplenomegaly, short stature, recurrent upper respiratory infections, hearing loss, hernias, dysostosis multiplex, joint contractures, and cardiac valve disease. Age of onset and disease severity vary greatly among individuals. Although MPS II involves a continuum of phenotypes, it has historically been described in two distinct forms. Individuals with the neuronopathic form of MPS II experience central nervous system (CNS) involvement, including developmental delay and progressive cognitive decline. In contrast, the CNS is not affected in the non-neuronopathic form of the disease. Heterozygous females typically do not show any symptoms of the disease [1].

Intravenous enzyme replacement therapy (ERT) with idursulfase is the only FDA-approved treatment for MPS II. Idursulfase has been shown to improve liver and spleen volumes, 6 min walk test (6MWT) distance, and forced vital capacity in pulmonary function testing (PFT) [2,3,4]. However, ERT cannot cross the blood–brain barrier (BBB) and therefore cannot prevent CNS involvement in the neuronopathic form of MPS II. Several therapies are currently under development, including brain-penetrant ERT and gene therapy. Hematopoietic stem cell transplant (HSCT) has also been tried in individuals with MPS II, but data are overall limited and conflicting [5,6,7].

MPS II was added to the Recommended Uniform Screening Panel (RUSP) in the United States in 2022, [8] and is currently screened for in 11 states. Newborn screening for MPS II began in Pennsylvania in 2023. Screening is also ongoing in several other countries, such as Taiwan, where it was initiated in 2015 [9]. In the era of early detection of MPS II through newborn screening, understanding the genotype has become increasingly important. Genotypic analysis helps distinguish between individuals who will develop the disease from those who merely carry genetic variants that result in low enzyme levels without causing clinical symptoms. Here, we report seven cases from four unrelated families identified through newborn screening, all sharing the same *IDS* variant (c.817C>T, p.Arg273Trp). We present and discuss their biochemical and clinical findings to elucidate the phenotypic consequences of this variant.

## 2. Materials and Methods

### 2.1. Taiwan Newborn Screening Program

Since 2015, a newborn screening program for MPS II has been in place in Taiwan. Between August 2015 and December 2024, a total of 713,933 newborns were screened using dried blood spots analyzed by tandem mass spectrometry. Of these, 252 infants with suspected MPS II were referred to MacKay Memorial Hospital for confirmatory testing. Diagnosis was confirmed through IDS enzyme activity assays in leukocytes, two-dimensional electrophoresis of urinary GAGs, quantitative analysis of dermatan sulfate, heparan sulfate, keratan sulfate, and chondroitin sulfate by liquid chromatography–tandem mass spectrometry (LC-MS/MS), and genetic analysis of *IDS* gene variants, as previously described [9].

### 2.2. Pennsylvania Newborn Screening Program

Newborn screening for MPS II in Pennsylvania was initiated on 1 July 2023. From 1 July 2023 to 30 June 2025, a total of 253,401 newborns were screened. Of these, 23 newborns were referred for confirmatory testing, and 5 of them were diagnosed with MPS II. The remaining cases were carriers (4), had pseudodeficiency alleles (9), or were unaffected (5). Newborn screening is performed through dried blood spot testing. Enzyme activity is measured by LC-MS/MS and is defined as the amount of product hydrolyzed by the enzyme in the reaction. Results are reported as µmol/L/h. Endogenous non-reducing ends (NREs) are measured as second-tier testing using LC-MS/MS. *IDS* sequencing is also performed as second-tier testing.

### 2.3. Clinical Cases

Medical records were reviewed to collect data on newborn screening results, biochemical testing, physical examinations, and imaging.

## 3. Results

### 3.1. Case 1

Case 1 is a male who was referred for evaluation at 28 days of life due to decreased iduronate-2-sulfatase activity level detected through Taiwan newborn screening. Enzyme activity was measured at 0.11 µmol/L/h (reference value ≥ 6.50) initially and 0.05 µmol/L/h (reference value ≥ 2.20) on repeat testing. He was born by spontaneous vaginal delivery at 39 5/7 weeks. There were no complications during the pregnancy. His initial physical examination was unremarkable. Confirmatory testing was sent and demonstrated low enzyme activity with increased urine GAGs (Table 1). Genetic testing identified a hemizygous variant in *IDS*: c.817C>T. An echocardiogram identified an atrial septal defect or a patent foramen ovale. Abdominal ultrasound, hand X-ray, and lumbosacral spine X-ray were all within normal limits. He was reassessed at 4.8 years of age. His development and growth were normal. Repeat echocardiogram showed a spontaneous closure of the previously identified defect. Abdominal ultrasound, hand X-ray, lumbosacral spine X-ray, and pelvis X-ray were again unremarkable. Enzyme activity was repeated and fell within normal limits (19.69 µmol/4 h/mg protein, reference value 12.89–131.83), but urine heparan sulfate was still elevated (41.56 µg/mL, reference value < 0.41).

### 3.2. Case 2

Case 2 is the older brother of Case 1. He was first assessed at 4.2 years of age and was found to share the familial *IDS* variant. His enzyme activity was significantly decreased, and elevated urine heparan sulfate was detected (Table 1). His physical examination was unremarkable. At his most recent follow-up at 8.7 years of age, he continued to demonstrate normal growth and cognition, without any signs of MPS II. Echocardiogram, abdominal ultrasound, hand X-ray, lumbosacral spine X-ray, and pelvis X-ray were normal. Enzyme activity had normalized upon repeated testing (21.5 µmol/4 h/mg protein, reference value 12.89–131.83), but urine heparan sulfate was still elevated (17.2 µg/mL, reference value < 0.41).

### 3.3. Case 3

Case 3 is a male who was referred for evaluation at 11 weeks of age following abnormal Taiwan newborn screening. Iduronate-2-sulfatase activity was decreased at 0.17 µmol/L/h (reference value ≥ 6.50) in the first sample, then 0.18 µmol/L/h and 0.27 µmol/L/h (reference value ≥ 2.20) in repeat samples. The pregnancy was uncomplicated, and he was born by spontaneous vaginal delivery at 40 6/7 weeks. His physical examination was unremarkable, and biochemical testing confirmed low enzyme activity and revealed increased urine GAGs (Table 1). He was found to be hemizygous for the c.817C>T variant in *IDS*. Echocardiogram revealed a thickened mitral valve. Additional workup, including abdominal ultrasound, hand X-ray, lumbosacral spine X-ray, and pelvis X-ray, were unremarkable. He was seen again for follow-up at 4 months of age, and there were no signs of MPS II. Repeat biochemical testing remained consistent with the diagnosis, with low enzyme activity (6.17 µmol/4 h/mg protein, reference value 12.89–131.83) and elevated urine GAGs (dermatan sulfate 0.9 µg/mL, reference value < 0.8; heparan sulfate 12.38 µg/mL, reference value < 0.41).

### 3.4. Case 4

Case 4 was referred for evaluation after Taiwan newborn screening at 10 weeks of age. His newborn screening demonstrated low iduronate-2-sulfatase activity: 0.08 µmol/L/h (reference value ≥ 6.50) in the first sample, then 0.17 µmol/L/h and 0.18 µmol/L/h (reference value ≥ 2.20) in repeat samples. He was born at 40 weeks following an uncomplicated pregnancy, and physical examination was normal. Confirmatory testing was consistent with a diagnosis of MPS II (Table 1), and the hemizygous *IDS* c.817C>T variant was identified. Echocardiogram, abdominal ultrasound, hand X-ray, lumbosacral spine X-ray, and pelvis X-ray were normal. At the time of his most recent assessment at 3.8 years of age, he remained asymptomatic, with normal growth and cognitive development. Imaging remained within normal limits. Repeat biochemical testing again showed decreased enzyme activity (1.56 µmol/4 h/mg protein, reference value: 12.89–131.83) and increased urine heparan sulfate (110.99 µg/mL, reference value < 0.41).

### 3.5. Case 5

Case 5 is a male who was referred for evaluation at 22 days of life after his Pennsylvania newborn screening demonstrated a low iduronate-2-sulfatase activity level (0.19 µmol/L/h, reference value ≥ 4.70) and elevated glycosaminoglycan ratio (5.52, reference value ≤ 1.84). *IDS* sequencing was performed as part of the newborn screening, and the hemizygous variant, *IDS* c.817C>T, was identified and reported as likely pathogenic. He was born by spontaneous vaginal delivery at 39 weeks following an uncomplicated pregnancy and is the third son of a non-consanguineous couple. Physical examination was unremarkable, and it was noted that he passed his newborn hearing screen. Confirmatory testing revealed decreased enzyme activity and increased GAGs in blood and urine (Table 1). Repeat testing at 3 months of age showed persistent elevation in urine GAGs (heparan sulfate 44 g/mol creatinine, reference range 0–5.28) and in urine NREs (UA-HexNAc-1S, #2 177.9 µmol/mol creatinine, reference range: 0–8; UA-HexN-UA-2S, 1100.8 µmol/mol creatinine, reference range: 0–9.7). Additionally, an abdominal ultrasound was performed to rule out hepatosplenomegaly and was unremarkable, except for the incidental finding of a 2 mm stone in the left kidney. An echocardiogram demonstrated low normal left ventricular function at 3 weeks of age, but normalized on repeat echocardiogram at 7 weeks of age. Cardiac evaluation was otherwise unremarkable. His neurodevelopmental assessment at 8 months of age demonstrated cognitive, language, and motor skills in the average to above average range.

### 3.6. Case 6

Case 6 is the older brother of Case 5 and was first assessed at 21 months of age after he was found to carry the familial *IDS* variant. He was born at 40 weeks. There were no complications during the pregnancy and perinatal period. He did not have any health concerns, and his growth and development were appropriate for his age. Physical examination was significant for right congenital trigger thumb. His iduronate-2-sulfatase activity level came back low, and GAGs were increased in both urine and blood (Table 1). Skeletal survey, abdominal ultrasound, and echocardiogram were unremarkable. Audiological evaluation demonstrated normal hearing. He underwent formal neurodevelopmental assessment, which showed skills in the average to above average range for his age.

### 3.7. Case 7

Case 7 is the maternal grandfather of Cases 5 and 6 and was assessed at 60 years old after he was found to carry the familial *IDS* variant. He had normal growth and development in childhood and graduated from university. His medical history is significant for a right inguinal hernia repair in his forties and bilateral Dupuytren contractures affecting his fifth fingers, which presented in his fifties. Physical examination was unremarkable aside from bilateral Dupuytren contractures. Namely, there were no coarse facial features or hepatosplenomegaly, and his height was normal (178 cm, 58th percentile). Subsequent testing showed low enzyme activity and elevated urine GAGs, while blood GAGs were normal (Table 1). Skeletal survey demonstrated age-related degenerative arthritis changes but was normal otherwise. Echocardiogram was normal. He underwent hearing testing, which revealed asymmetric mild to moderate sensorineural hearing loss in the right ear and mild hearing loss in the left ear.

## 4. Discussion

Seven individuals from four different familieswere found to share the *IDS* c.817C>T variant. Some information regarding Cases 1 to 4 was previously reported in different publications about the Taiwan newborn screening program [9,10,11], while this is the first study to report Cases 5 to 7. Biochemical testing demonstrated low enzyme activity and elevated urine GAGs in all individuals, consistent with a diagnosis of MPS II. However, these individuals did not exhibit typical clinical findings of MPS II, including the oldest, who was 60 years old. Case 3 was found to have a thickened mitral valve on echocardiogram, which can be seen in MPS II [1] but is a non-specific finding. No other findings consistent with MPS II were identified through physical examination and imaging. Similarly, Case 7 had an abnormal hearing test demonstrating mild-to-moderate sensorineural hearing loss. While conductive and sensorineural hearing loss occurs in most individuals with MPS II [1], alternative causes, such as presbycusis, might have contributed to the condition in this 60-year-old individual who did not display any other features of MPS II.

The *IDS* c.817C>T missense variant is absent from gnomAD [12]. Previous functional studies by Lin et al. showed significantly reduced IDS enzyme activity in the COS-7 cell construct expressing this variant, which was 2.2% of that observed in the wild-type construct [13]. This is consistent with the significantly reduced enzyme activity observed in our cohort. This variant has been reported once outside of the individuals included in this study. The other individual was identified in a newborn screening sample with significantly reduced IDS activity in a pilot study from Washington, USA. Clinical information is not available for this individual [14].

Pseudodeficiency for IDS is relatively common. Typically, individuals with pseudodeficiency have higher IDS activity compared to affected individuals and have normal urine GAGs. However, it has been shown that individuals with pseudodeficiency alleles may have mildly elevated urine GAGs and reduced enzyme activity, making it harder to determine disease status [15]. The individuals presented in this cohort all had significantly reduced enzyme activity during initial testing and persistently elevated urine GAGs at a level more suggestive of an attenuated form of the disease. Therefore, this does not fit the classical definition of pseudodeficiency, where only the in vitro enzyme activity is reduced [16]. In addition, Herbst et al. evaluated GAG biomarkers in newborn dried blood spot samples to distinguish between affected individuals, unaffected cases, and cases with pseudodeficiencies. They included a sample from Taiwan with the c.817C>T variant, which demonstrated GAG levels as high as in other MPS II individuals and clearly different from those with known pseudodeficiency alleles [17].

None of the individuals presented in this case series had been treated at the time of publication. Individuals from Taiwan are not eligible for treatment in the absence of symptoms [13]. Given the identification of an asymptomatic 60-year-old male relative, the decision was made not to initiate treatment in the individuals from Pennsylvania. ERT represents a non-negligible burden on families, with weekly intravenous infusions. There are also inherent risks to treatment, such as infusion-related reactions [18]. The decision to initiatetreatment can be challenging in the context of newborn screening for lysosomal storage diseases given the great variability in disease severity and progression. As newborns with MPS II typically do not exhibit any obvious signs of the disease in the first few weeks or months of life [1], data on genotype–phenotype correlation are highly valuable with regard to the necessity of treatment initiation.

Overall, our findings suggest that the *IDS* c.817C>T variant leads to a biochemical profile consistent with MPS II but lacks progressive clinical pathology. We hypothesize that the residual enzymatic function is sufficient to prevent the development of clinical manifestations. As newborn screening for MPS II becomes more widespread, it is anticipated that additional cases with this variant will be identified, providing further insights into its clinical significance. At present, initiation of treatment for individuals harboring the *IDS* c.817C>T variant should be guided by comprehensive clinical assessment, as current evidence does not support therapeutic intervention. Nonetheless, given that most individuals described here were still quite young, with five out of seven aged less than 5 years of age at the time of their last assessment, we recommend careful clinical monitoring to assess any signs of the disease over time.

Furthermore, as the implementation of newborn screening expands, it is likely that other previously unrecognized *IDS* variants with similar biochemical profiles but limited clinical manifestations will be discovered. Recognizing these cases will be crucial for refining treatment decisions and improving our understanding of genotype–phenotype correlations in MPS II. This evolving landscape underscores the importance of individualized clinical evaluation and the need for ongoing research to inform evidence-based management strategies.

## Figures and Tables

**Table 1:** Initial confirmatory testing and additional investigations.

Test (Units)	Individual 1 [Reference Value]	Individual 2 [Reference Value]	Individual 3 [Reference Value]	Individual 4 [Reference Value]	Individual 5 [Reference Value]	Individual 6 [Reference Value]	Individual 7 [Reference Value]
Age at referral (years)	0.1	4.2	0.2	0.2	0.1	1.8	60
Age at last assessment (years)	4.8	8.7	0.4	2.4	0.7	2.3	60
Enzyme activity	**0.2** [12.89–131.83 µmol/4 h/mg protein]	**0.41** [12.89–131.83 µmol/4 h/mg protein]	**4.82** [12.89–131.83 µmol/4 h/mg protein]	**4.74** [12.89–131.83 µmol/4 h/mg protein]	**1.58** [3.8–170.3 nmol/4 h/mL plasma]	**1.58** [3.8–170.3 nmol/4 h/mL plasma]	**1.58** [155–1082 nmol/4 h/mL plasma]
**Urine GAGs**							
Total (mg/mmol creatinine)	**77.3** [20.9–68.3]	16.73 [20.9–68.3]	**72.7** (20.9–68.3)	59.14 [20.9–68.3]	48.32 [0–53]	**24.68** [0–24.00]	**6.83** [0–6.50]
Dermatan sulfate	**7.39** [<0.80 µg/mL]	**5.48** [<0.80 µg/mL]	**0.96** [<0.80 µg/mL]	**1.18** [<0.80 µg/mL]	**18.77** [0–18.47 g/mol creatinine]	**8.00** [0–6.14 g/mol creatinine]	3.74 [0–4.59 g/mol creatinine]
Heparan sulfate	**1.83** [<0.41 µg/mL]	**24.9** [<0.41 µg/mL]	**17.89** [<0.41 µg/mL]	**40.38** [<0.41 µg/mL]	**96.17** [0–5.28 g/mol creatinine]	**72.57** [0–2.48 g/mol creatinine]	**17.61** [0–1.07 g/mol creatinine]
**Blood GAGs**							
Dermatan sulfate (nmol/L)	N/A	N/A	N/A	N/A	**253** [≤130]	67 [≤130]	51 [≤130]
Heparan sulfate (nmol/L)	N/A	N/A	N/A	N/A	**279** [≤95]	**134** [≤95]	37 [≤95]
*Urine NREs*							
UA-HexNAc-1S, #2 (µmol/mol creatinine)	N/A	N/A	N/A	N/A	**141.2** [0–8.0]	**93.2** [0–8.0]	**34.7** [0–8.0]
UA-HexN-UA-2S (µmol/mol creatinine)	N/A	N/A	N/A	N/A	**715.7** [0–9.7]	**438.9** [0–9.7]	**165.6** [0–8.0]
**Additional investigations**							
Abdominal US	Normal	Normal	Normal	Normal	Small kidney stone	Normal	N/A
Echocardiogram	ASD/PFO	Normal	Thick mitral valve	Normal	Normal	Normal	Normal
Skeletal survey	Normal	Normal	Normal	Normal	N/A	Normal	Age-related degenerative arthritis
Hearing test	Normal	Normal	Normal	Normal	Normal	Normal	Mild to moderate SNHL

Values outside of the reference range are indicated in bold. ASD—atrial septal defect; GAGs—glycosaminoglycans; NREs—non-reducing ends; PFO—patent foramen ovale; SNHL—sensorineural hearing loss; US—ultrasound. Urine GAGs were assessed at Greenwood Genetic Center for Individuals 5–7, blood GAGs were assessed at Mayo Clinic Laboratories, and urine NREs were assessed at the Children’s Hospital of Philadelphia.

## Data Availability

The original contributions presented in this study are included in the article. Further inquiries can be directed to the corresponding author(s).

## Abbreviations

The following abbreviations are used in this manuscript:

MPS	Mucopolysaccharidosis
GAG	Glycosaminoglycan
CNS	Central nervous system
ERT	Enzyme replacement therapy
FDA	U.S. Food and Drug Administration
RUSP	Recommended Uniform Screening Panel
LSD	Lysosomal storage disease
6MWT	6 min walk test
PFT	Pulmonary function tests
BBB	Blood–brain barrier
HSCT	Hematopoietic stem cell transplant
DS	Dermatan sulfate
HS	Heparan sulfate
KS	Keratan sulfate
CS	Chondroitin sulfate
LC-MS/MS	Liquid chromatography–tandem mass spectrometry
NRE	Non-reducing end
